# Vegetation restoration increases soil aggregate stability and aggregate-associated black carbon content in a karst rocky desertification area in southwestern Guangxi

**DOI:** 10.3389/fpls.2026.1784263

**Published:** 2026-03-19

**Authors:** Junzhi Chu, Kechao Huang, Qiumei Teng, Yuyi Shen, Yingjie Sun, Denan Zhang, Guangping Xu, Tao Ding, Liangliang Huang, Zhiwen Tan, Yunhuang Luo

**Affiliations:** 1 Guangxi Key Laboratory of Plant Conservation and Restoration Ecology in Karst Terrain, Guangxi Institute of Botany, Guangxi Zhuang Autonomous Region and Chinese Academy of Sciences, Guilin, China; 2 College of Environmental Science and Engineering, Guilin University of Technology, Guilin, China; 3 Guangxi Key Laboratory of Functional Phytochemicals and Sustainable Utilization, Guangxi Institute of Botany, Guangxi Zhuang Autonomous Region and Chinese Academy of Sciences, Guilin, China

**Keywords:** black carbon, carbon sequestration, karst, restoration, soil aggregates, vegetation

## Abstract

Vegetation restoration is a critical strategy for combatting rocky desertification in the karst region. Soil black carbon (BC) contributes to the soil organic carbon (SOC) pool and plays an important role in carbon storage. However, the impact of vegetation restoration on BC accumulation under consistent geological (karst lithology) and climatic conditions is not well known. The distribution of BC in aggregates and its role in SOC also require clarification. We investigated the impact of vegetation restoration on soil aggregate stability and the micro-distribution of SOC and BC across a successional gradient (secondary forest, shrubland, farmland, and wasteland) in a karst rocky desertification area. Results showed that vegetation restoration significantly enhanced soil structural stability, with secondary forest and shrubland exhibiting the highest mean weight diameter (MWD) and geometric mean diameter (GMD). Compared with farmland and wasteland, the proportions of water-stable macroaggregates (> 0.25 mm) in secondary forest and shrubland areas increased to over 73%. SOC, BC and TCa concentrations were highest in secondary forest topsoil and decreased with increasing soil depth, while the BC/SOC ratio increased with depth, indicating the selective preservation of recalcitrant carbon in deeper soil layers. Macroaggregates are mostly responsible for SOC and BC accumulation in each land-type category. A significant positive correlation exists between aggregate stability, TCa and SOC and BC stocks (*p* < 0.05), suggesting a “carbon dual-protection” mechanism: woody vegetation promotes soil aggregation via enhanced root-soil interactions and organic inputs, while high soil calcium enhances the stability of large aggregates and mineral protection ability. These findings indicate that robust vegetation cover promotes the physical occlusion of BC within stable macroaggregates, protecting it from degradation and erosion. Therefore, prioritizing restoration efforts to the secondary forest stage can improve the long-term carbon sequestration potential and soil erosion resistance in fragile karst ecosystems.

## 1 Introduction

Soils, the largest terrestrial carbon reservoir, store over 70% of the planet’s organic carbon—a mass greater than that contained in all living biomass and the atmosphere combined (Köchy et al., 2015; Parras-Alcantara et al., 2015). Even minor fluctuations in the soil organic carbon (SOC) pool can significantly impact atmospheric CO_2_ concentrations and global climate dynamics (Lehmann and Kleber, 2015). The formation of soil aggregates begins with the agglomeration of finer structural components like silt and clay. These primary aggregates then combine into larger structural units, ultimately building up the overall soil architecture (Jahn et al., 2006). Key mechanisms in this process include cohesive forces, the bonding of inorganic substances, cementing action of organic matter, and formation of organo-mineral complexes (Onweremadu et al., 2007; Cheng et al., 2015; Deng et al., 2018). Plant functional traits, such as root architecture and exudates, act as biological binding agents that initiate the formation of macro-aggregates (Rui et al., 2022).

The stability of soil aggregates directly determines soil physical, chemical, and biological properties, plays a key role in maintaining soil fertility, promotes water infiltration, improves aeration, and enhances soil erosion resistance (Mikha and Rice, 2004). The physical protection of SOC by soil aggregates also affects long-term carbon sequestration (Tang et al., 2016). Through physical encapsulation, soil aggregates limit oxygen diffusion and microbial accessibility, thereby extending the mean residence time of carbon in the soil (Dungait et al., 2019). Within the SOC pool, black carbon (BC)—a continuum of combustion residues ranging from partially charred biomass to highly condensed soot—is an important component of the inert SOC pool (Schmidt and Noack, 2000). BC is a key player in the global carbon cycle (Coppola et al., 2022), with an estimated global annual production of 62–294 Tg, of which 80%–90% is deposited directly into the soil (Gao et al., 2023). BC accumulates carbon is considered to be a critical strategy for climate change mitigation (Sharma and Mishra, 2022) and constitutes a significant component of the “missing carbon” in the Earth’s carbon budget (Forbes et al., 2006; Preston and Schmidt, 2006). This is attributed to BC’s ability to increase carbon sequestration (Xu et al., 2018), mitigate greenhouse gas emissions (Shrestha et al., 2010), and enhance soil fertility (Gao et al., 2023).

BC dynamics have been explored in forests (Ohlson et al., 2009), farmland (Zhang et al., 2015), wetland (Wang et al., 2014), and urban areas (Ji et al., 2017). These studies have consistently demonstrated that BC can increase stable SOC stocks. Crucially, the stability of BC is dependent both on its chemical recalcitrance and its physical location within the soil matrix. BC preferentially accumulates in microaggregates and occluded particulate organic matter fractions (Brodowski et al., 2006). This physical encapsulation within aggregates shields BC from microbial degradation and acts as a stabilization mechanism (Xu et al., 2018). However, how BC accumulation and soil aggregate stability are coupled in the process of vegetation restoration, particularly in calcium-rich karst environments, warrants further investigation.

The karst region in southwest China is one of the largest continuous karst areas in the world (Jiang et al., 2014; Zhao et al., 2021). Here, shallow soil layers and discontinuous vegetation cover render the ecosystem highly susceptible to rocky desertification—a process of land degradation that involves severe soil erosion and bedrock exposure (Wang et al., 2004; Jiang et al., 2014). To mitigate this degradation, large-scale vegetation restoration projects such as the “Grain for Green” program have been implemented to convert sloping farmlands into forests, shrublands, or wastelands (Wang et al., 2023). Notably, the karst ecosystem is characterized by a calcium-rich geochemical background. The interaction between abundant calcium ions (Ca^2+^) and soil organic carbon acts as a unique stabilization mechanism, often referred to as cation bridging (Bronick and Lal, 2005; Rowley et al., 2018). Therefore, further clarifying how vegetation restoration influences this biological-geochemical coupling process is crucial for assessing the long-term soil carbon sequestration effectiveness of these regional ecological engineering projects. Vegetation restoration is generally considered to enhance soil quality by increasing organic matter input from litterfall and root exudates, which act as binding agents to promote the formation of water-stable macroaggregates (Yang et al., 2024). While many studies have confirmed that vegetation restoration increases total SOC stocks (Hu and Lan, 2020; Li et al., 2024), the specific mechanisms governing the stabilization of recalcitrant carbon fractions, particularly BC, within soil aggregates in these calcium-rich, erosion-prone karst soils remain poorly understood.

Previous research on BC has focused on its content or source identification in agricultural soil (Rumpel et al., 2006; Zhang et al., 2015), and has often overlooked its micro-distribution within soil aggregate fractions in karst areas. Although effects of vegetation restoration on soil aggregates and SOC have been reported (Tang et al., 2016; Hu and Lan, 2020),it remains unclear how the transition from degraded land to restored vegetation alters the physical protection of BC and whether the coupling of BC with macroaggregates contributes to the enhanced erosion resistance of calcium-rich karst soils.

To address these knowledge gaps, we examine soil samples from four typical land-type categories in a karst rocky desertification area. Specific research objectives include: (1) evaluating the effects of vegetation restoration on soil aggregate size distribution and stability; (2) quantifying the accumulation and distribution characteristics of SOC, BC and TCa within aggregate size classes; and (3) elucidating the mechanisms by which vegetation restoration enhances the physical protection of BC and the stability of soil structure.

**Figure 1:**
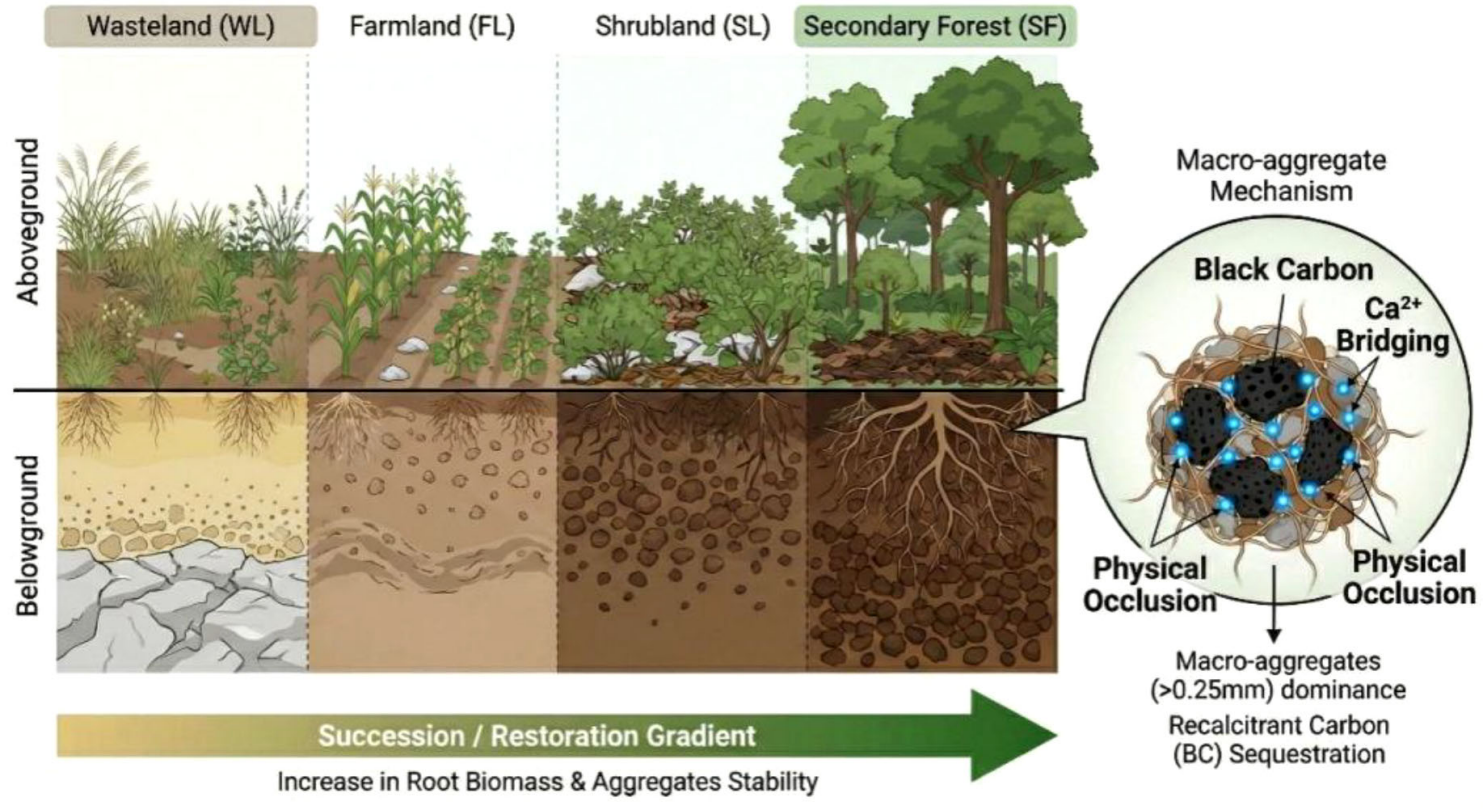
Conceptual framework illustrating vegetation restoration driving soil aggregate stability and carbon sequestration mechanisms. WL, Wasteland; FL, Farmland; SL, Shrubland; SF, Secondary Forest; BC, Black Carbon; SOC, Soil Organic Carbon; Ca^2+^, Calcium ions; >0.25 mm: Water-stable macro-aggregates.

Based on the aggregate hierarchy theory and the geological background in the karst region, we propose a conceptual framework to guide this investigation (**Figure 1**). We hypothesize that the transition from wasteland to secondary forest drives the development of extensive root networks. The roots acting in synergy with the calcium-rich geochemical background (Ca^2+^ bridging), facilitate the physical occlusion of BC within stable macro-aggregates, creating a ‘carbon dual-protection’ system. This research provides theoretical insights into the long-term carbon sequestration potential of ecological restoration strategies in fragile karst ecosystems, emphasizes the importance of BC accumulation in stabilizing SOC across different land-use types.

## 2 Materials and methods

### 2.1 Study area

The study was performed in a representative area (Guohua Town, Pingguo City, Guangxi Province (107°22′40–25′30″E, 23°22′30″–24′00″N; elevation 110–570 m)) of the karst peak-cluster depression landscape in southwest China. The region experiences a subtropical monsoon climate and mean annual temperature of 19.1–22.0 °C. Approximately 70% of rainfall occurs between May and August, and the mean annual precipitation is ~1500 mm. Shallow brown limestone soils (calcareous lithosol) derived from carbonate rocks predominate, and extensive rock is exposed. In degraded areas, vegetation cover is sparse and rocky desertification is severe.

**Figure 2:**
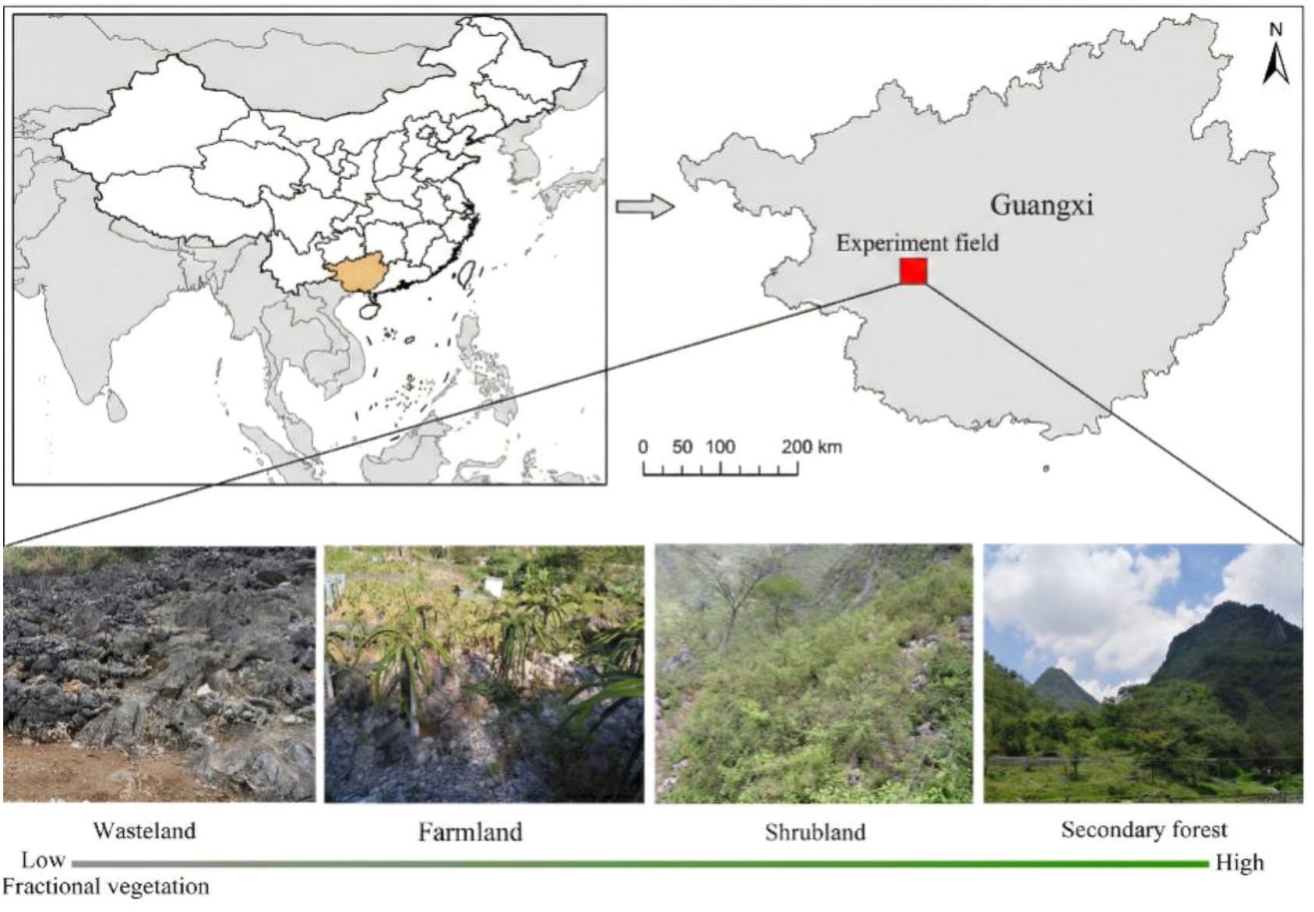
Study area location map, with land-type categories associated with vegetation restoration.

Four typical land-type categories that represent a distinct gradient of fractional vegetation cover (from sparse vegetation and bare soil to a dense canopy) were selected: Wasteland (WL), Farmland (FL), Shrubland (SL), and Secondary Forest (SF) (**Figure 2**). WL sites (vegetation coverage ~26%) are croplands that have been abandoned for ~17 years and are dominated by sparse herbaceous vegetation such as *Artemisia argyi*, *Bidens pilosa*, *Cynodon dactylon*, and *Miscanthus sinensis*. FL sites (vegetation cover ~44%) have been cultivated for over 100 y with crops including maize (*Zea mays*), soybean (*Glycine max*), and pitaya (*Hylocereus undatus*). SL sites (restored for ~39 years; vegetation cover ~73%) are dominated by species such as *Alchornea trewioides*, *Cipadessa cinerascens*, and *Vitex negundo* (Lv et al., 2018). SF sites (restored for ~80 y; vegetation cover ~85%) are characterized by woody species including *Zenia insignis*, *Melia azedarach*, *Apodytes dimidiata*, and *Choerospondias axillaris*. All sampling sites were located at south-facing mid-slope positions on gradients from 13–17°, sharing a consistent geological background of continuous carbonate rock (dolomite) parent materials. Research was performed in an open area. No specific sampling permits were required because of established cooperative relationships with local residents.

### 2.2 Soil sampling

Soil sampling was performed in November 2020. In each of the four land categories, three representative replicate plots (50 × 50 m) were established. To ensure sample representativeness while preserving soil structure, a spatially stratified sampling strategy was used. Within each plot, five sub-sampling points were randomly selected in an “S” shape. At each point, undisturbed soil blocks were carefully collected using a spade from 0–10, 10–20, 20–30, and 30–40 cm depth intervals. The five sub-samples from the same soil layer within each plot were mixed to form a single composite sample.

During sampling, visible coarse roots and gravel were removed. Care was taken to prevent soil compression to preserve natural aggregates. Samples were placed into rigid plastic boxes to protect macroaggregates from disruption during transport. Upon arrival at the laboratory, fresh soil samples were gently broken apart along natural fracture planes and passed through an 8-mm sieve, strictly following the protocol required for subsequent wet-sieving analysis (Wang et al., 2020).

### 2.3 Sample analyses

#### 2.3.1 Soil aggregate fractionation

Water-stable aggregates were fractionated using a modified wet-sieving method (Six et al., 1998). Fresh soil samples passed through an 8-mm sieve were placed on the top of a nested sieve set with apertures of 2.000, 0.250, and 0.053 mm. To simulate rapid wetting and minimize slaking, the samples were immersed in deionized water and soaked for 15 min. Sieving was then performed using an aggregate analyzer (TPF-100) at a vertical oscillation frequency of 30 cycles min^−1^ for 30 min. This process yielded four aggregate size fractions: large macroaggregates (> 2 mm), small macroaggregates (0.25–2 mm), microaggregates (0.053–0.25 mm), and a silt–clay fraction (< 0.053 mm). Aggregates retained on each sieve were carefully washed into containers and oven-dried at 60 °C to constant weight. Dried samples were weighed to calculate the mass percentage of each size fraction and stored for carbon analysis. We define macroaggregates as the sum of > 2 mm and 0.25–2 mm fractions (> 0.25 mm).

#### 2.3.2 Physico-chemical properties

SOC contents of the bulk soil and each aggregate fraction were determined using a Total Organic Carbon Analyzer (Shimadzu 5000A, Japan). BC contents were quantified using the chemical-thermal oxidation method of (Lim and Cachier, 1996). Briefly, 3 g of soil sample was treated with 3 mol·L^−1^ HCl and 10 mol·L^−1^ HF to remove carbonates and silicates. The residue was then subjected to thermal oxidation in a muffle furnace at 375 °C for 24 h to remove non-BC organic carbon. The final residue was analyzed using an elemental analyzer (Flash EA 1112, Thermo Finnigan, Italy) to determine BC concentration. Refer to Bao (Bao, 2000) method to determine the following indicators: bulk density (BD) using the ring knife method; soil total nitrogen(TN) were measured using a VarioEL CHN elemental analyzer (Elementar Analysensysteme GmbH, Germany); soil total phosphorus (TP) content was determined using the molybdenum antimony blue colorimetry method after extracting with the HClO_4_ and H_2_SO_4_; the total potassium (TK) content was measured using the NaOH melting method combined with flame photometry; the available potassium (AK) was determined with CH_3_COONH_4_; available nitrogen (AN) was measured using the alkaline diffusion method, while available phosphorus (AP) was determined with NaHCO_3_ extraction and measured by colorimetry method. Soil total calcium content was digested with HNO_3_-HClO_4_-HF and determined by atomic absorption spectrophotometry.

#### 2.3.3 Calculating aggregate stability

Soil aggregate stability was evaluated using Mean Weight Diameter (MWD), Geometric Mean Diameter (GMD), and Fractal Dimension (D), in accordance with the following equations (Yang et al., 2024):


$$MWD\;=\sum_{i=1}^{n}\omega_{i}{\bar{x}}_{i}$$

$$GMD\;=\exp(\sum_{i=1}^{n}\omega_{i}\ln{\left(\bar{x}\right)}_{i})$$

D was calculated based on the fractal model of particle size distribution (Millan et al., 2003):


$$D=3-log[\frac{m_{\left(i<x_{i}\right)}}{m_{t}}]/log[\frac{x_{i}}{x_{max}}]$$

where ${\bar{x}}_{i}$ is the mean diameter of the *i*-th aggregate size fraction (mm), $\omega_{i}$ is the mass fraction of aggregates in the *i*-th size fraction, ${\bar{x}}_{\max}$ is the mean diameter of the largest aggregate fraction (mm), m(_i <_ ${\bar{x}}_{i}$) represents the cumulative dry weight of aggregates with a diameter smaller than ${\bar{x}}_{i}$ (g), and m_t_ is the total dry weight of the soil sample (g).

### 2.4 Statistical analysis

Experimental data were compiled and preliminarily processed using Microsoft Excel 2019. Statistical analyses were performed using SPSS 19.0 software. Data normality was verified by Shapiro–Wilk tests. A two-way analysis of variance (ANOVA) was performed to evaluate the main effects of land-use type and soil depth, as well as their interactions, on basic soil physicochemical properties (**Table 1**). For soil aggregate stability and carbon fractions, given the significant interaction effects observed, one-way ANOVA followed by Tukey’s HSD *post-hoc* tests was applied to compare differences among land-use types within each soil layer to ensure detailed resolution. Relationships and dominant factors driving variations in soil structure and carbon sequestration were identified by Pearson correlation analysis and Principal component analysis (PCA) using Origin 2019. Random Forest models were employed to identify and rank the primary drivers of soil aggregate stability (MWD) and black carbon accumulation.

**Table 1:** Soil physical and chemical properties in different land use types.

Vegetation	Depth (cm)	TN (g·kg^-^1)	TP (g·kg^-^1)	TK (g·kg^-^1)	AN (mg·kg^-^1)
SF	0-10	4.20 ± 0.04aA	1.59 ± 0.03aA	4.58 ± 0.03aA	426.14 ± 4.18aA
	10-20	3.56 ± 0.15bA	1.37 ± 0.06bA	4.18 ± 0.04bA	354.86 ± 11.63bA
	20-30	2.88 ± 0.14cA	1.09 ± 0.06cA	3.09 ± 0.12cA	221.49 ± 22.04cA
	30-40	1.96 ± 0.09dA	0.75 ± 0.10dA	2.16 ± 0.05dA	107.32 ± 5.39dA
SL	0-10	2.50 ± 0.21aB	1.41 ± 0.04aB	4.20 ± 0.07aB	304.53 ± 5.97aB
	10-20	2.06 ± 0.07aB	1.20 ± 0.05bA	3.93 ± 0.08aA	245.12 ± 18.31bB
	20-30	1.70 ± 0.15bB	1.06 ± 0.05bA	3.30 ± 0.19bA	193.71 ± 7.82cA
	30-40	1.18 ± 0.21cB	0.67 ± 0.12cA	2.06 ± 0.06cA	104.04 ± 7.00dA
FL	0-10	2.18 ± 0.13aB	1.22 ± 0.03aC	3.05 ± 0.06aC	199.82 ± 1.29aC
	10-20	1.77 ± 0.08bC	1.06 ± 0.05bB	2.29 ± 0.14bB	155.71 ± 9.32bC
	20-30	1.14 ± 0.08cC	1.03 ± 0.06bA	2.03 ± 0.07bB	112.26 ± 9.28cB
	30-40	0.99 ± 0.04cB	0.75 ± 0.10cA	1.24 ± 0.17cB	93.41 ± 7.97cA
WL	0-10	1.82 ± 0.15aC	0.77 ± 0.05aD	1.20 ± 0.05aD	118.47 ± 5.80aD
	10-20	1.36 ± 0.09bD	0.61 ± 0.03aC	1.05 ± 0.08aC	91.04 ± 3.35bD
	20-30	1.11 ± 0.06bC	0.47 ± 0.04cA	0.68 ± 0.06bC	78.09 ± 3.69cC
	30-40	0.97 ± 0.08cB	0.37 ± 0.03cB	0.35 ± 0.07cC	37.30 ± 4.42dB
**Two-way ANOVA**	**F(Vegetation)**	551.12***	251.91***	2059.76***	953.36***
	**F(Depth)**	294.68***	207.89***	809.32***	764.14***
	**F(Interaction)**	14.77***	7.95***	37.88***	80.26***
	**Depth** (cm)	**AP (mg·kg^-^1)**	**AK (mg·kg^-^1)**	**BD (g·cm^-^1)**	**TCa (g·kg^-^1)**
**Vegetation** SF	0-10	5.27 ± 0.06aA	92.53 ± 0.44aA	1.07 ± 0.03bC	27.78 ± 0.29aA
	10-20	5.03 ± 0.09aA	80.58 ± 1.79bA	1.21 ± 0.05cC	21.85 ± 0.30bA
	20-30	4.29 ± 0.19bA	57.05 ± 5.43cA	1.33 ± 0.05aB	15.35 ± 0.33cA
	30-40	3.23 ± 0.08cA	34.34 ± 2.72dA	1.38 ± 0.03aC	11.31 ± 0.23dA
SL	0-10	4.33 ± 0.17aB	82.64 ± 1.40aB	1.18 ± 0.04bC	23.47 ± 0.24aB
	10-20	3.99 ± 0.13aB	68.85 ± 1.20bA	1.21 ± 0.04bC	17.24 ± 0.11bB
	20-30	3.28 ± 0.14bB	48.54 ± 2.34cB	1.41 ± 0.05aA	12.34 ± 0.21cB
	30-40	1.85 ± 0.19cB	29.11 ± 1.43dB	1.48 ± 0.04aB	9.44 ± 0.23dB
FL	0-10	2.97 ± 0.13aC	40.72 ± 1.74aC	1.25 ± 0.03cB	19.37 ± 0.55aC
	10-20	2.10 ± 0.07bC	31.55 ± 0.82bB	1.33 ± 0.04cB	13.21 ± 0.08bC
	20-30	1.77 ± 0.08cC	21.66 ± 1.25cC	1.46 ± 0.05bA	10.07 ± 0.06cC
	30-40	1.24 ± 0.09dC	16.30 ± 0.83dC	1.59 ± 0.05aA	7.57 ± 0.11dC
WL	0-10	2.11 ± 0.13aD	14.00 ± 1.54aD	1.37 ± 0.04cA	14.57 ± 0.24aD
	10-20	1.55 ± 0.26bD	10.32 ± 0.62bC	1.43 ± 0.01bA	10.82 ± 0.20bD
	20-30	1.21 ± 0.09bD	6.78 ± 0.24cD	1.48 ± 0.03bA	8.60 ± 0.16cD
	30-40	0.88 ± 0.14cD	2.92 ± 0.55dD	1.52 ± 0.04aA	5.16 ± 0.10dD
**Two-way ANOVA**	**F(Vegetation)**	1160.46***	2260.04***	71.14***	3248.86***
	**F(Depth)**	401.99***	831.03***	130.73***	6353.18***
	**F(Interaction)**	15.63***	82.73***	4.51***	118.09***

TN, Total nitrogen; TP, Total phosphorus; TK, Total potassium; AN, Available nitrogen; AP, Available phosphorus; AK, Available potassium; BD, Bulk density; TCa, Total calcium; SF, Secondary forest; SL, Shrubland; FL, Farmland; WL, Wasteland. Values are mean ± standard deviation (n=3). F-values are derived from two-way ANOVA. *** *p* < 0.001. Different lowercase letters within the same column indicate significant differences (*p* < 0.05) among soil layers within the same type, while different uppercase letters indicate significant differences (*p* < 0.05) among types within the same soil layer.

Bold text indicates the parameters of the two-way analysis of variance (ANOVA).

## 3 Results

### 3.1 Soil physical and chemical properties

Two-way ANOVA results for soil physicochemical properties indicated that land-use type, soil depth, and their interaction had significant effects (*p* < 0.001, **Table 1**). Specifically, the contents of TN, TP, TK, AN, AP, AK, and TCa were all significantly higher in secondary forest compared with other land-use types. The BD was higher in wasteland than in other land-use types (**Table 1**) (*p* < 0.05). As the depth of the soil layer increased, various chemical properties gradually decreased, while the soil bulk density tended to increase.

### 3.2 Aggregate stability

**Figure 3:**
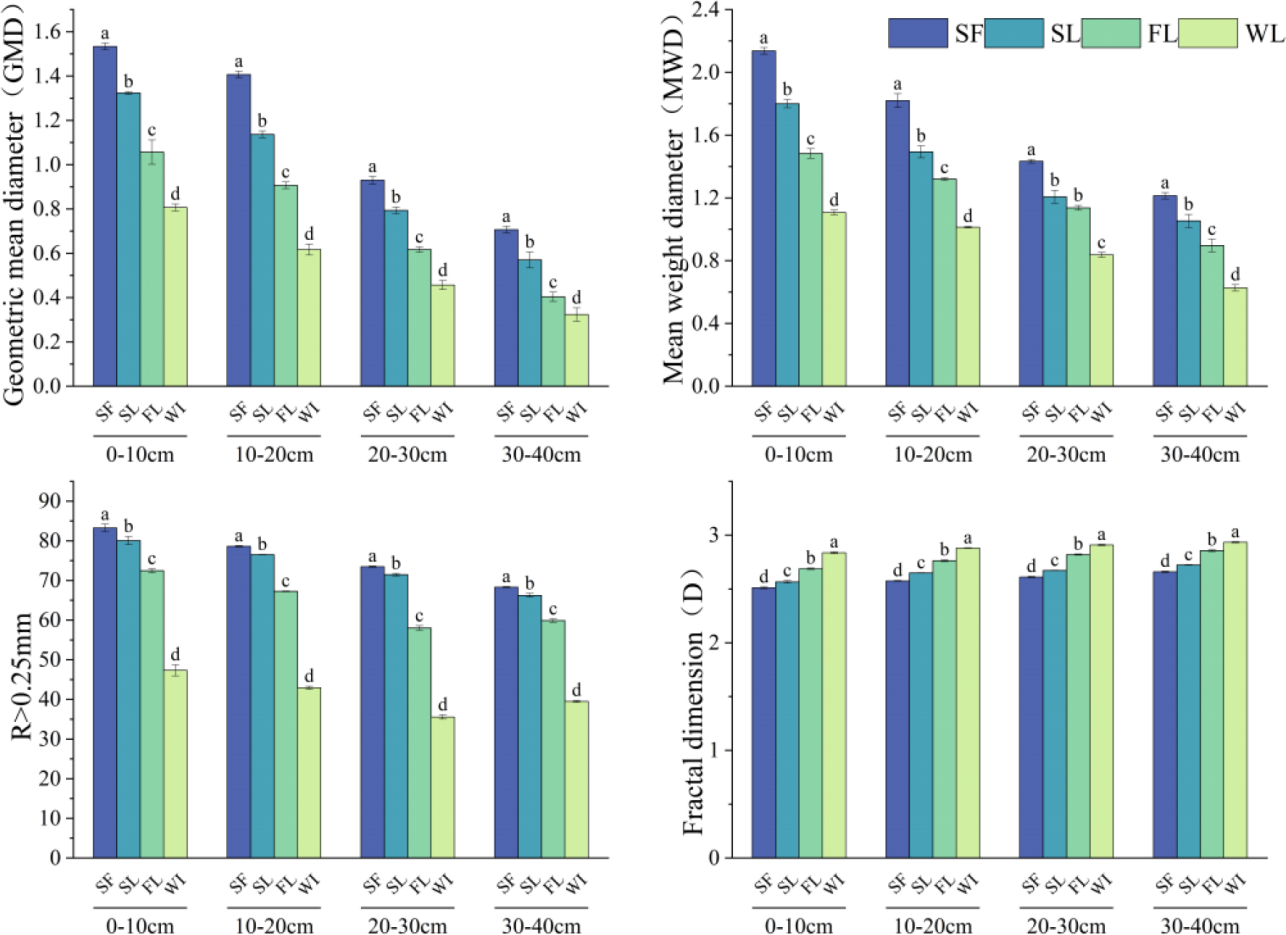
Aggregate stability by land-type category: SF, Secondary forest; SL, Shrubland; FL, Farmland; WL, Wasteland. Different lowercase letters indicate significant differences between land-type categories (*p* < 0.05).

Soil aggregate stability results are presented in **Figure 3**. The primary stability indicators—MWD, GMD, and the content of water-stable macroaggregates (R > 0.25 mm)—consistently ranked SF > SL > FL > WL across all depths; D had the opposite order (WL > FL > SL > SF). Stability indicators generally decreased with increased soil depth, with all metrics peaking from 0–10 cm; MWD and GMD were highest in SF (compared with WL, the MWD and GMD values under SF in the 0–10 cm layer increased by 92.8% and 88.9%, respectively).

D significantly increased with depth in all land-type categories; from 0–40 cm, it was consistently highest in WL and lowest in SF. Stability parameters differed significantly in topsoil (0–20 cm) among land-use types (*p* < 0.05): with restoration generally enhancing stability across the 0–40 cm profile.

### 3.3 Aggregate size distribution by land-type category

**Figure 4:**
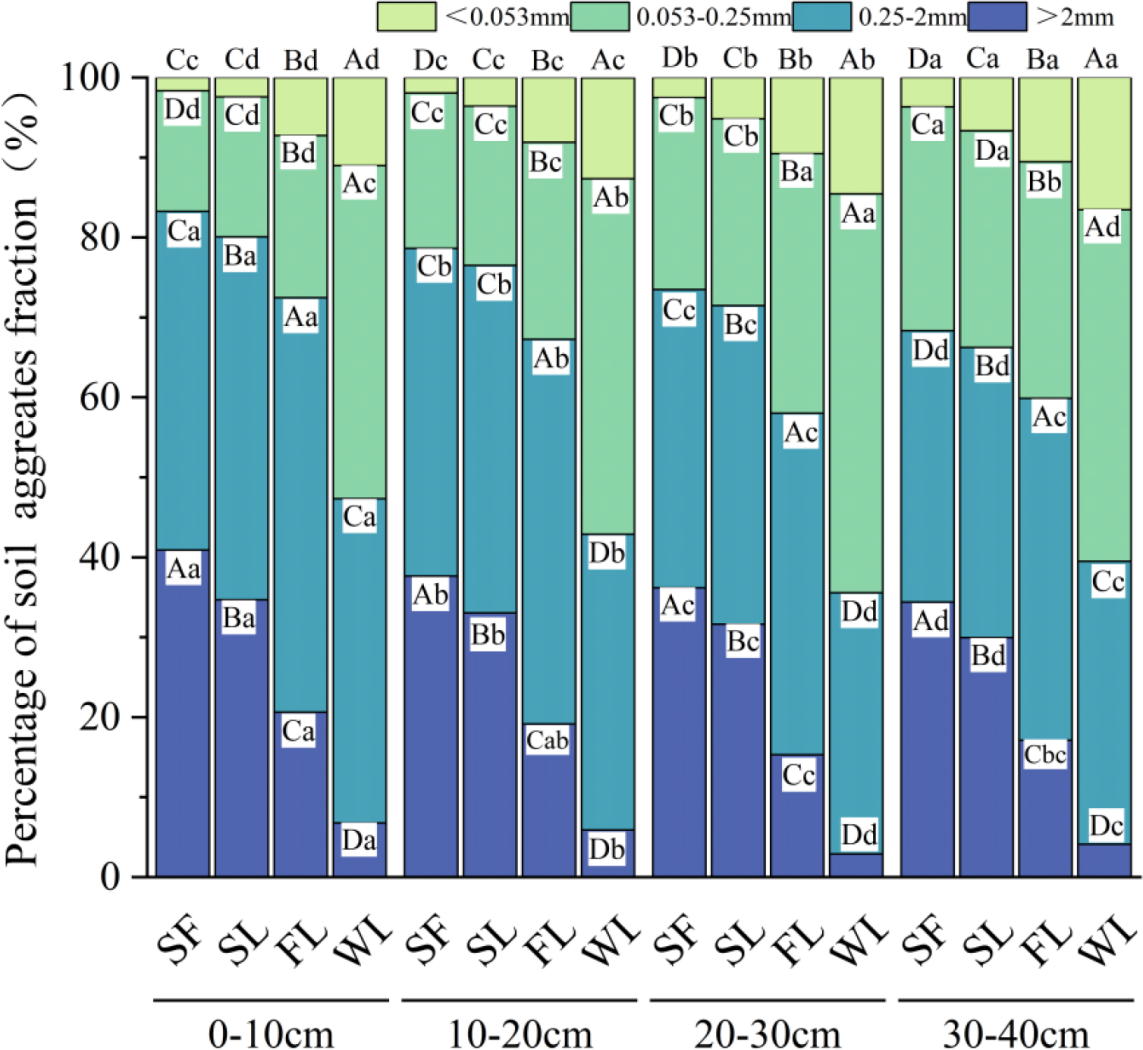
Distribution of soil water stability aggregates by land-type category: SF, Secondary forest; SL, Shrubland; FL, Farmland; WL, Wasteland. Lower-case letters indicate significant differences (*p* < 0.05) between layers in the same land-type category; upper-case letters indicate significant differences (*p* < 0.05) between land-type categories within a layer.

The distribution patterns of soil water-stable aggregates were consistent among land-type categories for each soil layer (from 0–40 cm; **Figure 4**). Specifically, SF and SL had the highest macroaggregate proportions (>73%). This proportion was significantly higher than that of FL (64.40%) and WL (41.32%; *p* < 0.05). Conversely, the proportion of microaggregates (< 0.053 mm) trended in the opposite direction: SF < SL < FL < WL (*p* < 0.05). Macroaggregate contents decreased significantly from the 0–10 cm layer to the 30–40 cm layer across land-type categories, while microaggregate proportions had the inverse trend (*p* < 0.05). Consequently, the proportion of macroaggregates significantly increased, and that of microaggregates significantly decreased in the 0–40 cm soil layer as the stage of vegetation restoration advanced from the WL to SF (*p* < 0.05). The proportions of macroaggregate contents also decreased, and those of microaggregates increased with increased soil depth in each land-type category.

### 3.4 Vertical distributions of SOC, BC and TCa by land-type category

**Figure 5:**
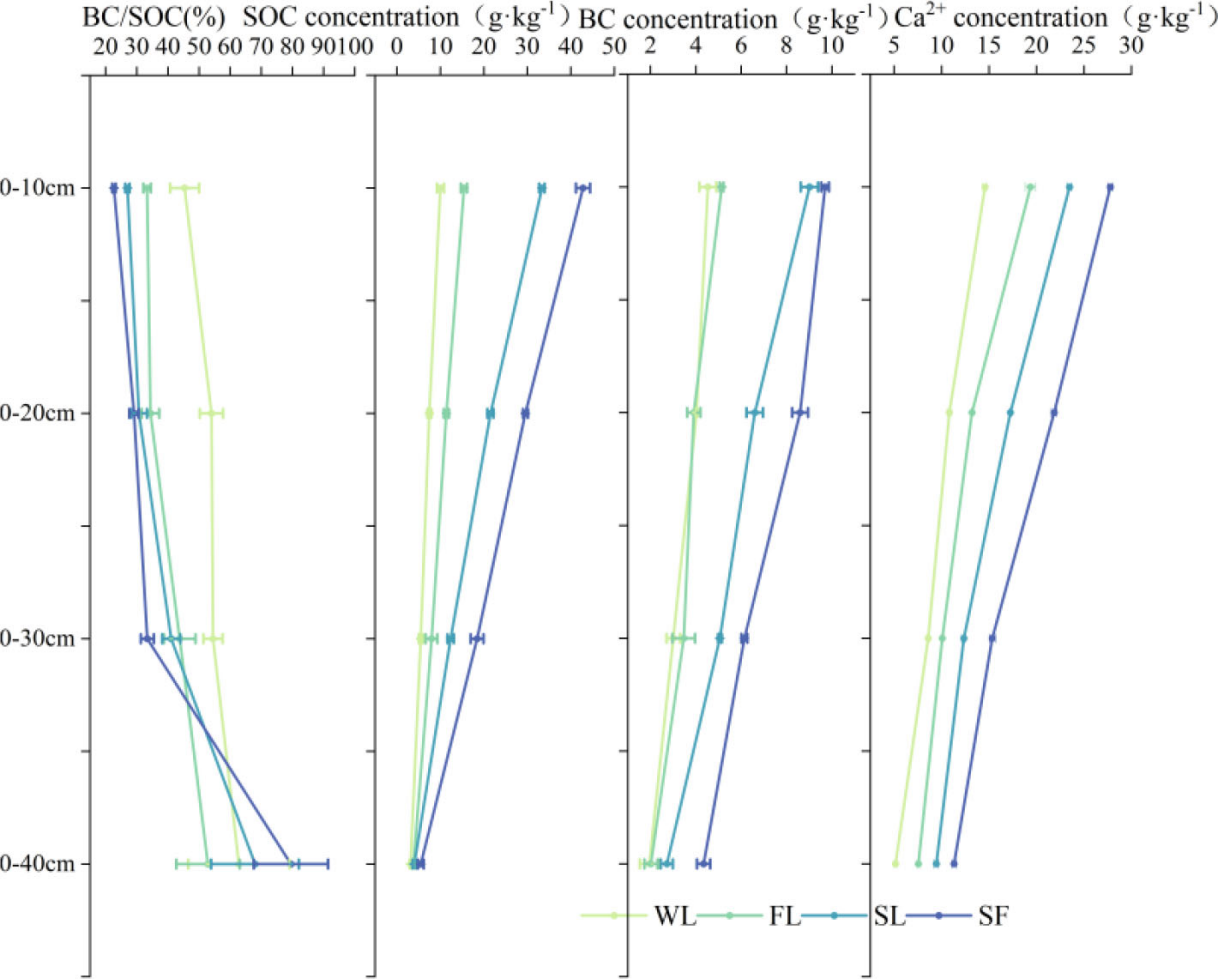
Variation in SOC (g***·***kg^-1^), BC (g***·***kg^-1^), BC/SOC ratio (%) and Ca (g***·***kg^-1^) with soil depth by land-type category: WL, Wasteland; FL, Farmland; SL, Shrubland; SF, Secondary forest.

SOC, BC and TCa concentrations exhibited a consistent pattern of decreasing with increasing soil depth across all vegetation types (**Figure 5**). Concentrations of both were highest in topsoil (0–10 cm), and lowest were from 30–40 cm. For each layer, the overall magnitude followed an SF > SL > FL > WL order. In the topsoil (0–10 cm), SOC, BC, and TCa concentrations were consistently highest under SF and lowest under WL (*p* < 0.05). Specifically, SOC content in SF was more than four times that of WL. The concentrations of all three attributes decreased significantly with soil depth, with the most pronounced reduction occurring between the 0–10 cm and 10–20 cm layers.

TCa concentration was significantly higher in the SF topsoil (27.78 g**·**kg^−1^) compared to the WL (14.57 g**·**kg^−1^).The decrease in both SOC, BC and TCa concentrations was most pronounced between the 0–10 cm and 10–20 cm layers in each land-type category. For instance, the SOC concentration in SF dropped by 13.23 g kg^−1^ in this interval, whereas the reduction in WL was 2.55 g kg^−1^.

In contrast to the trend in absolute concentrations, the BC/SOC ratio generally increased with soil depth, peaking in the 30–40 cm layer across all land-use types. In the topsoil (0–10 cm), the ratio was lowest under SF and highest under WL. However, this ratio generally increased with soil depth across all land-use types, peaking in the 30–40 cm layer. This vertical pattern contrasts with the absolute concentrations, indicating the selective preservation of BC in deeper soils.

### 3.5 Contributions of aggregate OC, BC, and TCa to their respective total pools

**Figure 6:**
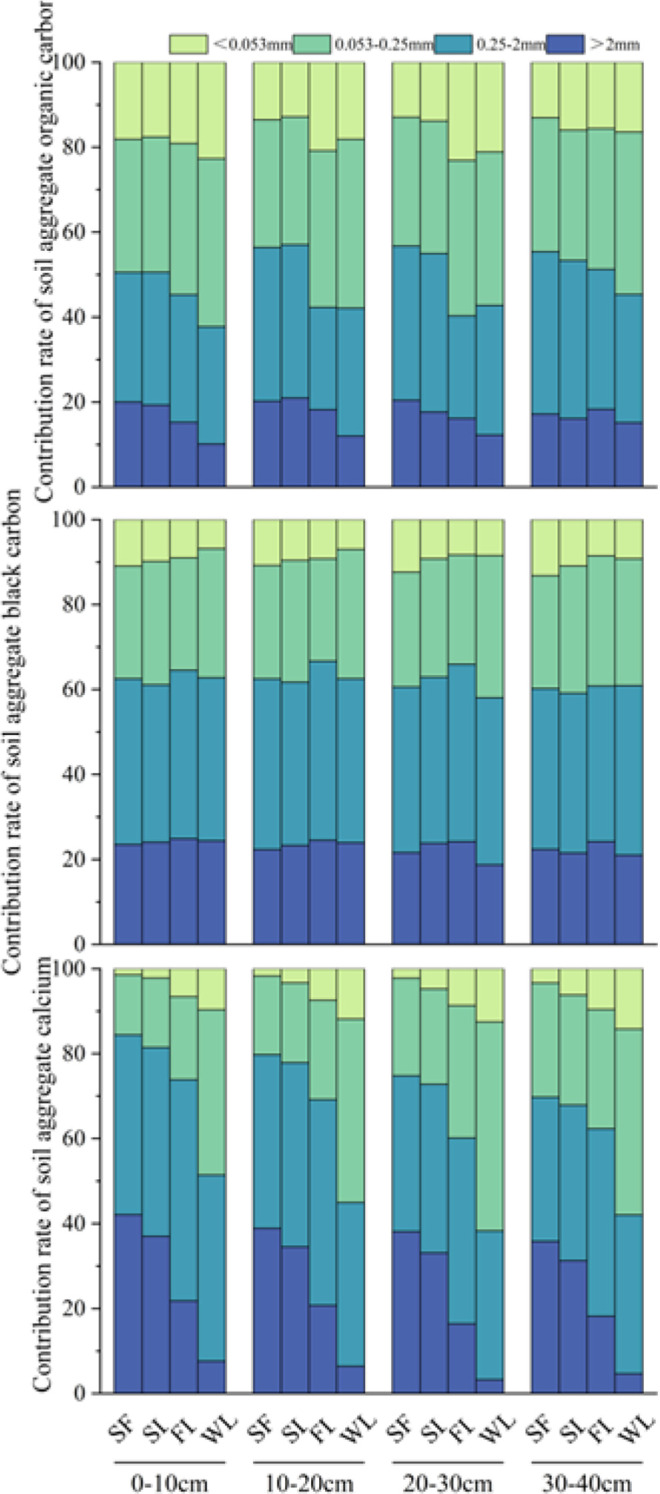
Contributions of aggregate SOC (upper), BC (middle) and TCa (lower) by land-type category: SF, Secondary forest; SL, Shrubland; FL, Farmland; WL, Wasteland.

Contributions of aggregate organic carbon to SOC accumulation by land-type category are shown in **Figure 6** (upper). From 0–10 cm, SF and SL had notably higher organic carbon contribution rates for macroaggregates (> 0.25 mm) than FL and WL, dominating the total SOC pool. Conversely, the contribution of the silt–clay fraction (< 0.25 mm) to SOC accumulation was highest in WL among land categories. Although the aggregate organic-carbon contribution rates of macroaggregates decreased with increased soil depth (to 30–40 cm), SF and SL had consistently higher levels than either other land category. Therefore, following vegetation recovery to SL and SF stages, the topsoil (0–10 cm) accumulation of SOC was mainly attributed to macroaggregates. This indicates that robust vegetation cover promotes organic carbon sequestration within stable soil structures.

The contributions of aggregate BC to BC accumulation are shown in **Figure 6** (middle). From 0–40 cm, the contributions of macroaggregates (> 0.25 mm) to BC accumulation were greatest among all particle sizes in each land-type category. Unlike SOC, the distribution of BC did not fluctuate markedly with land category; even in FL and WL, macroaggregates accounted for the majority of BC accumulation, with only a slightly higher contribution from the silt–clay fraction (< 0.053 mm) compared with either other land category.

The contributions of aggregate calcium to TCa accumulation are shown in **Figure 6** (lower). Although the aggregate calcium contribution rates of macroaggregates decreased with increased soil depth (to 30–40 cm), SF, SL and FL all had notably higher calcium contribution rates for macroaggregates (> 0.25 mm) than WL category, dominating the total calcium pool. Conversely, the contribution of the silt–clay fraction (< 0.25 mm) to TCa accumulation was highest in WL from 0—40cm soil layer.

### 3.6 Pearson correlation analysis of soil aggregate SOC, BC and Ca contents, and aggregate proportion and stability

**Figure 7:**
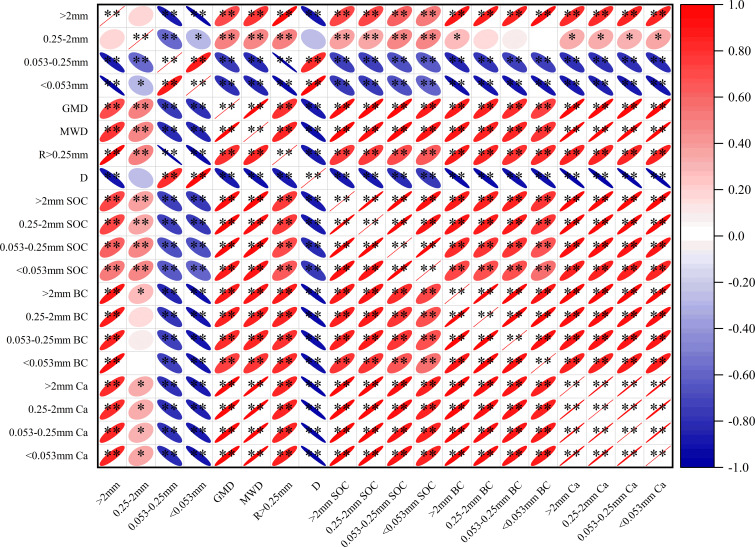
Pearson correlations between soil aggregate stability, aggregate BC, Ca and carbon content of aggregates. MWD, Mean weight diameter; D, fractal dimension; GMD, geometric mean diameter; Ca, calcium; SOC, soil organic carbon; BC, black carbon.*p≤ 0.05; **p ≤ 0.01. Red represents positive correlation, green represents negative correlation. The darker the color and the larger the elliptical area, mean the stronger the correlation.

Soil MWD, GMD, and R > 0.25 mm correlated positively with the > 2 mm fraction, and negatively with 0.053–0.25 mm and < 0.053 mm fractions and D (all *p* < 0.01) (**Figure 7**). Moreover, D correlated negatively with the > 2 mm fraction, WMD, GMD, and R > 0.25 mm, and positively with the < 0.25 mm fraction (all *p* < 0.01). The correlation between aggregate-associated SOC and BC was positive across all particle sizes (*p* < 0.01). The correlation between aggregate-associated Ca, SOC and BC were positive across all particle sizes (*p* < 0.01), highlighting a degree of spatial synchronization among organic carbon, recalcitrant carbon, and mineral binding components. Specifically, SOC and BC contents in all aggregate fractions correlated positively with stability parameters (WMD, GMD, and R > 0.25 mm and the > 2 mm fraction), and negatively with D and the < 0.25 mm fractions (all *p* < 0.01). For instance, the > 2 mm SOC correlated strongly with the > 2 mm BC, WMD, and GMD. Similarly, Ca content in all aggregate fractions correlated positively with stability parameters (WMD, GMD, and R > 0.25 mm and the > 2 mm fraction), and negatively with D (all *p* < 0.01).

### 3.7 Principal component analysis of soil aggregate proportions, and aggregate stability and associated SOC, BC and Ca

**Figure 8:**
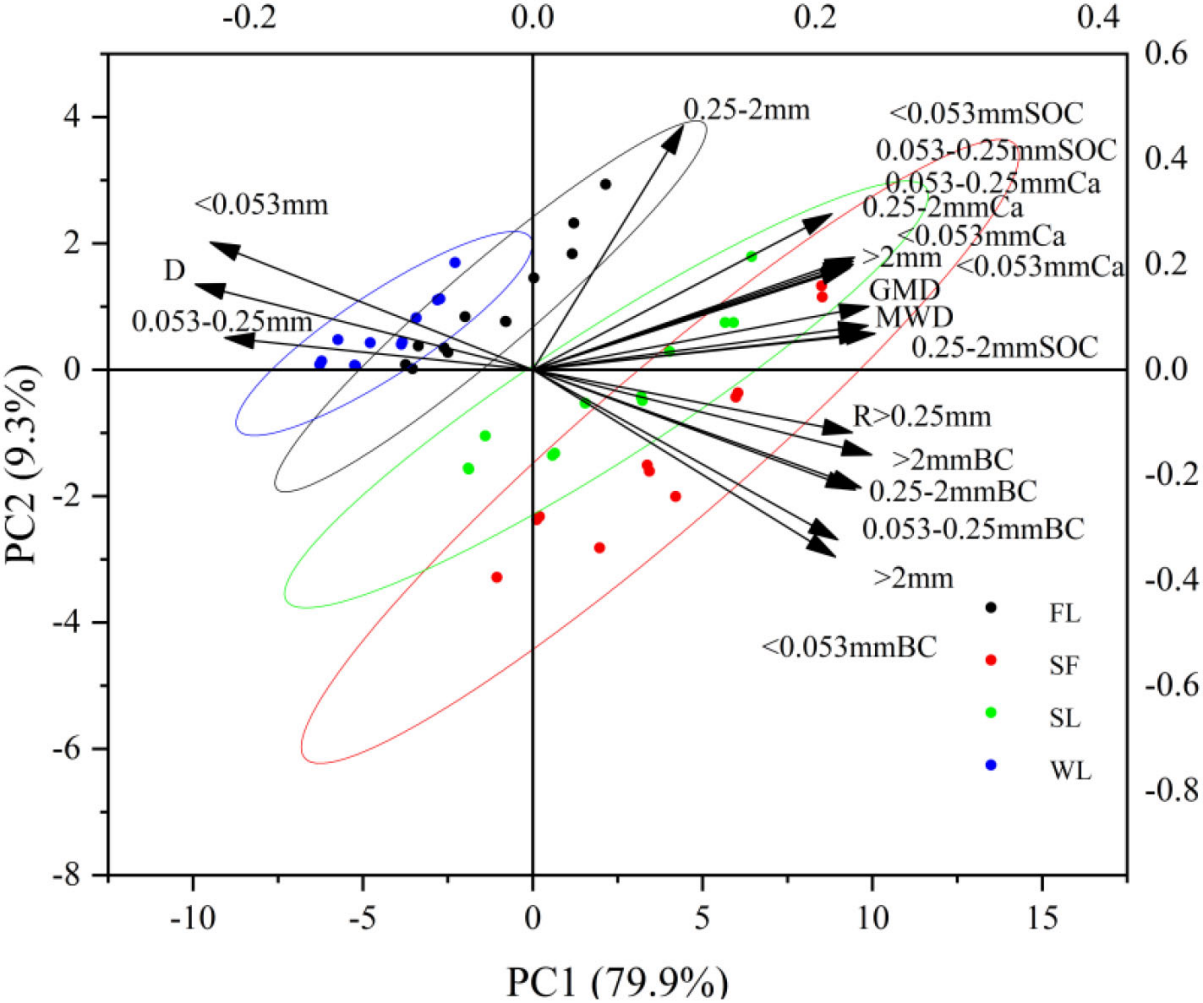
Principal component analysis of aggregate fractions, stability, aggregate-associated soil organic content, black carbon and calcium. MWD, Mean weight diameter; D, fractal dimension; GMD, geometric mean diameter; TCa, calcium; SOC, soil organic carbon; BC, black carbon. Blue represents wasteland (WL), black represents farmland (FL), green represents shrubland (SL), and red represents secondary forest (SF) land-type category, respectively.

Multivariate relationships among soil aggregate stability, particle size distribution, carbon and calcium components by land-type category are depicted in **Figure 8**. The first two principal components (PC1 and PC2) together explain 89.2% of the total variance, with PC1 accounting for 79.9% and PC2 for 9.3%. Notably, the variance explained by PC1 is approximately 8.6× that of PC2, indicating that PC1 captures the major variability in soil properties. Along the PC1 axis, the sampling sites within land-type categories exhibited distinct clustering patterns: SF and SL clustered in a positive direction in PC1 and FL and WL were concentrated in a negative direction. Aggregate stability indices (MWD, GMD, R > 0.25 mm), macroaggregate fractions (> 2 mm and 0.25–2 mm), and aggregate-associated SOC, BC and Ca contents were heavily loaded on the positive side of PC1, aligning closely with the distribution of SF and SL. Conversely, D and the fine particle fraction (< 0.053 mm) were associated with the negative direction, corresponding to FL and WL sites. This distribution suggests that aggregate-associated SOC, BC and Ca contents increased synchronously with aggregate stability.

### 3.8 Drivers of aggregate stability and black carbon accumulation

**Figure 9:**
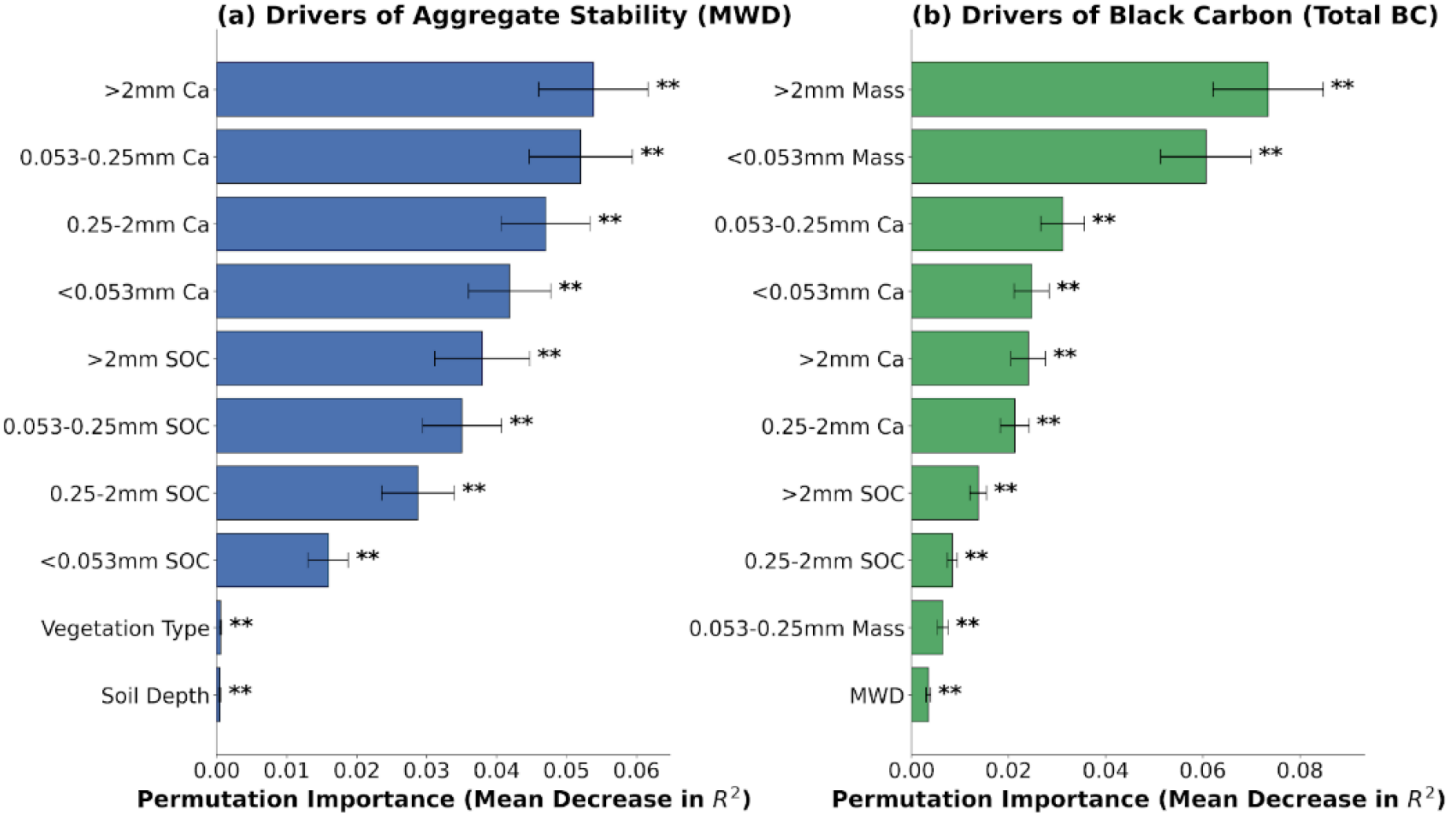
Random forest mean predictor importance (increase in MSE) of aggregate mass fractions, associated chemical properties (SOC, Ca) for **(a)** aggregate stability (MWD) and **(b)** total black carbon (Total BC). Both models demonstrated high explanatory power with R^2^ > 0.99. Significance levels are indicated by asterisks: *p < 0.05, **p < 0.01. Error bars represent the standard deviation of the permutation importance.

Random forest models were employed to identify the key drivers of soil aggregate stability (MWD) and BC accumulation. Both models demonstrated exceptionally high explanatory power, with R^2^ > 0.99. For aggregate stability, the calcium content within large macroaggregates (>2 mm Ca) and the organic carbon associated with large macroaggregates (>2 mm SOC) were identified as the most critical predictors (**Figure 9a**). Specifically, >2 mm Ca exhibited the highest permutation importance (p < 0.01), followed by the significant contribution of >2 mm SOC (p < 0.01). Regarding BC accumulation, the mass fraction of large macroaggregates (>2 mm Mass) was the primary driver (**Figure 9b**), causing the largest increase in model error when permuted (p < 0.01). The aggregate stability index (MWD) and >2 mm SOC were also significant predictors (p < 0.01). In contrast, the direct influences of vegetation type and soil depth were negligible in both models.

## 4 Discussion

### 4.1 Effects of vegetation on soil aggregate distribution and stability

Consistent with previous findings (Zhang et al., 2019b; Wang et al., 2023; Zhang et al., 2024), soil aggregate stability significantly improved following land restoration with vegetation. This indicates that restoring vegetation (artificial planting and natural restoration) can enhance soil structure in karst rocky desertification areas, compared to farmland and wasteland. MWD and GMD reveal aggregate stability to be highest in the SF land-type category and lowest in WL. In the topsoil (0–10 cm) layer in SF, MWD was 92.8% higher than in WL, and GMD was 88.9% higher. Conversely, D exhibited the opposite trend (WL > FL > SL > SF; **Figure 3**). This indicates that agricultural reclamation activities or insufficient vegetation cover in FL and WL can damage soil macroaggregates.

Many studies have suggested that an increase in aggregate stability during restoration using vegetation is associated with increased organic matter content (Tisdall and Oades, 1982; Six et al., 2004; Brodowski et al., 2006) because organic matter acts as a cementing agent that promotes the formation and stabilization of soil aggregates. Our PCA (**Figure 8**) reveals SF and SL sites to be strongly associated with high aggregate stability and carbon content, whereas those in WL and FL sites were associated with structural fragmentation (high D values). The correlation between the proportion of aggregates > 0.25 mm (R > 0.25 mm) and MWD and GMD values was positive, but negative with D (all *p <* 0.01). This suggests that as vegetation recovers to SL and SF levels, the accumulated macroaggregates directly enhance geometric and weight-based soil stability and improve soil erosion resistance, consistent with other studies (An et al., 2013; Zhang et al., 2019b; Xiao et al., 2020; Abbas et al., 2021).

Aggregates play an important role in soil structure, and their size distribution and stability affect various factors such as soil porosity, water retention, permeability, and resistance to erosion (Amézketa, 1999). Our results indicate that vegetation significantly affects the particle size distribution of soil water-stable aggregates. During the vegetation restoration process (from WL and FL to SL and SF), the content of aggregates > 0.25 mm in soils increased, and that of aggregates < 0.25 mm decreased (both *p* < 0.05). Specifically, SF and SL had the highest proportions of macroaggregates (> 0.25 mm), exceeding 73% (**Figure 4**). Because soil aggregate stability increases when the proportion of aggregates > 0.25 mm is high, this proportion is a key indicator of soil aggregate stability (Zhang et al., 2019a). This indicates that microaggregates gradually cement to form macroaggregates and stabilize soil structure in secondary forests and shrub land use types, compared to farmland and wasteland.

Aggregate formation and stabilization involves complex processes (Six et al., 2004) that depend on a variety of biological and abiotic factors (such as the abundant Ca2+ in karst regions) associated with plant properties (e.g., roots and vegetation type) and soil properties (e.g., organic matter and microbiological activity) (Bronick and Lal, 2005). Compared with FL and WL, the SF and SL stages are characterized by more extensive root systems and abundant litter inputs. The physical enmeshment by roots and the secretion of organic exudates promote the binding of soil particles, thereby facilitating the flocculation of microaggregates into stable macroaggregates. The increase in soil aggregate stability following vegetation restoration in this karst rocky desertification area is consistent with results of other studies (An et al., 2013; Zhang et al., 2019a; Dou et al., 2020).

### 4.2 Vegetation restoration enhances carbon sequestration potential

Land restoration using vegetation significantly enhances the sequestration capacity of SOC and BC. SOC, BC and Tca contents followed the order of SF > SL > FL > WL at all soil depths (**Figure 5**). Specifically, in the 0–10 cm soil layer, the SOC content under SF reached 42.78 g kg−1; we attribute this to the long-term accumulation of biomass burning residues and their subsequent integration into the soil matrix. This trend is consistent with (Zhan et al., 2013), who reported vegetation restoration to significantly increase SOC in the Loess Plateau through root and litter inputs. The SF consistently had the greatest absolute SOC and BC concentrations across all depths, indicating that demonstrating robust vegetation cover significantly and positively affected SOC accumulation, which mostly occurs in the topsoil.

Beyond absolute content, the BC/SOC ratio provides insights that improve our understanding of the composition and stability of the accumulated carbon pool. Although BC content increased with vegetation restoration stage, its proportion to total SOC reflects a dynamic equilibrium between fresh carbon input and recalcitrant carbon retention. In restored ecosystems with high vegetation coverage (such as SF and SL sites), the substantial input of fresh organic matter acts as a “diluent” for BC in surface soils; thus, although BC accumulates, the BC/SOC ratio does not increase excessively (Rumpel et al., 2006; Yu et al., 2012). However, this ratio typically increases in deeper soils. This vertical trend is attributed to the selective preservation of BC, as labile SOC components are mineralized and decomposed by microorganisms, while the chemically stable BC is preserved, leading to subsoil enrichment (Li et al., 2021). In summary, the BC/SOC ratio consistently showed an inverse vertical pattern to absolute concentrations, with the strongest differentiation between land-type categories occurring in topsoil; it was lowest in SF, indicating that SOC turnover in SF was slow and highly inert, which is conducive to its accumulation.

The strong correlation between SOC and BC (**Figure 7**) suggests a synchronous accumulation mechanism. The soil aggregate-associated SOC and BC correlated with aggregate stability (*p* < 0.01); when the content of the > 2 mm fraction was high, the soil MWD, GMD, and reserves of SOC and BC increased, and D decreased. This indicates that macroaggregation promotes carbon sequestration and structural stability through soil BC. (Llorente et al., 2010) noted that the maintenance of BC proportions in different land-type categories implies that an equilibrium exists between inputs and outputs, and that BC, like SOC, may receive physical protection within improved soil structures. Soil restoration from WL to SF increased total carbon stocks and shifted the carbon pool toward a more recalcitrant and stable composition through BC accumulation, reducing the turnover rate of sequestered carbon effectively (Forbes et al., 2006). Relatively speaking, regardless of the vegetation restoration stage, macroaggregates served as the primary reservoir for BC physical storage. This suggests that BC exhibits a strong affinity for larger soil structures and is highly sensitive to anthropogenic disturbances such as fire and agricultural cultivation.

### 4.3 Coupling of Aggregates and BC: stabilization mechanisms

The distribution of SOC within aggregates reveals how land restoration by revegetation can enhance the carbon sink stability. In SF and SL land-type categories, macroaggregates (> 0.25 mm) were the primary carriers for SOC accumulation (**Figure 6**). However, in WL, SOC was mainly associated with the silt–clay fraction. This shift indicates a key stabilization mechanism: physical protection via occlusion. Organic matter occluded within aggregates (the occluded fraction) represents a protected carbon pool capable of resisting rapid biodegradation (Liu et al., 2024). Consistent with this, our random forest analysis identified the mass fraction of large macroaggregates (>2 mm Mass) as the unequivocal primary driver of total BC accumulation (**Figure 9b**). This provides compelling evidence that physical occlusion within macroaggregates serves as the primary shelter for sequestering recalcitrant carbon against degradation. The distribution of aggregate-associated TCa is spatially synchronized with SOC and BC content, macro-aggregates serving as the primary reservoirs for both components in SF and SL (**Figure 6**). This indicates that vegetation restoration not only drives the accumulation of SOC and BC, but also facilitates the enrichment of calcium within stable macro-aggregates. These findings provide direct evidence for the biogeochemical coupling of carbon and calcium, suggesting that the calcium-rich karst environment reinforces the physical protection of carbon through the stabilization of the macro-aggregate architecture. The Ca and the enrichment of mineral binding agents within macro-aggregates is linked to the simultaneous accumulation of soil organic carbon and black carbon (**Figure 7**), thereby reinforcing the structural integrity of karst soils through a synergistic stabilization mechanism Crucially, the random forest model identified >2 mm Ca and >2 mm SOC as the paramount predictors of aggregate stability (**Figure 9a**). This underscores that calcium-mediated cationic bridging is the primary mechanism stabilizing large macroaggregates in this karst ecosystem, acting as the fundamental constraint on soil structure. In SF soils, the formation of these macroaggregates establishes a physical barrier that restricts oxygen diffusion and microbial contact, thereby extending the mean residence time of encapsulated carbon. Therefore, improving aggregate stability by restoring vegetation can enhance soil erosion resistance and SOC and BC sequestration capacities.

BC exhibited a strong affinity for macroaggregates across all land-type categories. Even in soils with agricultural activities (FL and WL), macroaggregates accounted for the majority of BC accumulation. This indicates that BC may serve as a stable nucleus for aggregate formation. (Sun et al., 2016) reported a considerable proportion of BC in typical Changbai Mountain forest soils to occur in aggregate light fractions, contributing to soil structural stability. Furthermore, BC possesses a highly aromatic structure, providing intrinsic chemical stability, including surface oxidation leading to coordination bonding, strong adsorption of organo-mineral complexes, and enhanced microbial cementation (Sun et al., 2020). The porous structure and high specific surface area of BC provide abundant adsorption sites for mineral particles and organic matter, initiating the formation of micro-aggregates which then develop into macro-aggregates (Jien and Wang, 2013; Liu et al., 2023).The significant positive correlations between aggregate-associated Ca and BC fractions across all particle sizes provide direct evidence for this synergistic accumulation (**Figure 7**).

The high stability of Amazonian Dark Earths (Terra Preta) was largely attributed to the chemical recalcitrance of BC and its interaction with soil minerals (Glaser and Birk, 2012). We report the coupling of BC with macroaggregates to likely create a “dual-protection” mechanism: the inherent chemical inertness of BC (Forbes et al., 2006) combined with the physical encapsulation provided by the improved soil structure under SF and SL. This synergistic effect ensures that restored forests store more carbon, and store it in a form that is highly resistant to degradation. The specific geochemical context of the calcium-rich karst environment likely reinforces this stabilization. Given that aged BC surfaces often develop negative charges (e.g., carboxyl groups), Ca^2+^ ions can effectively bridge these organic particles with negatively charged soil mineral colloids (Rowley et al., 2018). In this high-calcium karst environment, the abundance of Ca^2+^ acts as a persistent geochemical stabilizer. The vegetation restoration triggers the coupling process by providing the necessary organic binding agents (roots/exudates) that interact with this pre-existing calcium matrix (Bronick and Lal, 2005). This has been validated in our PCA results (**Figure 8**). This cation-mediated bonding promotes the flocculation of microaggregates into water-stable macroaggregates and strengthens the connection between BC and the soil matrix (Liang et al., 2006). Consequently, in karst ecosystems, BC is stabilized by physical occlusion, chemical recalcitrance, and geochemical cementation driven by the high calcium background. This makes it exceptionally resistant to erosive forces.

### 4.4 Limitations and future perspectives

Although we provide significant insights into the effects of the stage of vegetation restoration on aggregate stability and carbon sequestration (SOC and BC) in the karst region, we acknowledge several limitations. First, the limitations of the “space-for-time” substitution method. We infer the temporal dynamics of vegetation restoration by comparing land-type categories (WL, SL, GL, FL) spatially. While this is a standard approach in ecological studies, it assumes that the initial soil conditions and parent materials were identical across all sites. In the heterogeneous karst landscape, high spatial variability in rock outcrops and soil depth may introduce uncertainties that a long-term longitudinal monitoring study could better resolve. We didn’t consider how does “time” affect macroaggregate formation in the absence of vegetation? Future research could consider long-term *in-situ* monitoring to quantify precise rates of carbon accumulation. Second, uncertainty exists in BC source apportionment and deep transport. While we quantify the distribution of BC across aggregate sizes, we did not differentiate the specific sources of BC (biomass burning vs. fossil fuel deposition) using isotopic signatures (δ^13^C) or molecular markers (BPCA). Additionally, karst regions are characterized by unique dual-structure hydrological processes (surface and underground leakage). Because we limited sampling to the 0–40 cm profile, we may have missed the deep vertical migration of dissolved or colloidal BC through limestone fissures (soil leakage)—an important pathway of carbon loss in karst environments. Further investigation could extend to deeper soil profiles and include isotopic tracing to fully close the carbon budget in this fragile ecosystem. Finally, although the chemical-thermal oxidation (CTO) method used in this study is a standard protocol for isolating recalcitrant carbon, it may inadvertently capture fractions of non-pyrogenic, acid-resistant organic carbon, such as condensed tannins and lignin. This could potentially lead to an overestimation of strictly defined pyrogenic carbon. Future research could benefit from integrating spectroscopic techniques (e.g., ^13^CNMR) or molecular marker analyses (e.g., BPCA) to cross-verify BC concentrations and sources.

## 5 Conclusions

This study elucidates the mechanisms linking vegetation restoration, soil aggregate stability, and black carbon (BC) sequestration in karst rocky desertification areas. The main findings are summarized as follows:

### 5.1 Vegetation restoration improves structure

The transition from wasteland and farmland to shrubland and secondary forest shifts soil dominant aggregates from microaggregates to water-stable macroaggregates (> 0.25 mm), significantly improving structural stability.

### 5.2 Carbon sequestration mechanism

Macroaggregates act as the primary carriers for SOC and BC. A “dual-protection” mechanism was identified: calcium availability governs aggregate stability via chemical cementing, while physical occlusion within macroaggregates is the prerequisite for long-term BC preservation.

### 5.3 Ecological implications

BC functions as both a recalcitrant carbon pool and a structural nucleus for aggregation. Therefore, restoration strategies should prioritize establishing deep-rooted woody communities (Secondary Forest stage) to maximize this aggregate-carbon-calcium coupling, and by minimizing agricultural planting reclamation activities and converting wasteland to forests, or to restore degraded land to forests type can reduce the loss of soil carbon (including BC), thereby enhancing long-term carbon sinks and erosion resistance in fragile karst ecosystems, may also offer a sustainable pathway to mitigate the effects of climate chang.

## Data Availability

The raw data supporting the conclusions of this article will be made available by the authors, without undue reservation.
